# Oxidant-templating fabrication of pure polypyrrole hydrogel beads as a highly efficient dye adsorbent†

**DOI:** 10.1039/c9ra00209j

**Published:** 2019-02-18

**Authors:** Xi Ye, Qingchi Xu, Jun Xu

**Affiliations:** Department of Physics, Research Institute for Biomimetics and Soft Matter, Fujian Provincial Key Laboratory for Soft Functional Materials, Xiamen University Xiamen 361005 P. R. China xujun@xmu.edu.cn; Jiujiang Research Institute of Xiamen University Jiujiang 332000 P. R. China; Shenzhen Research Institute of Xiamen University Shenzhen 518057 P. R. China

## Abstract

Polypyrrole (PPY) is a type of dye adsorbent with good adsorbing capability. Fabrication of PPY with a porous architecture, though technically challenging, can further enhance its dye adsorbing capability due to the tremendous increase of surface area. In this manuscript, an oxidant-templating strategy was developed to fabricate pure PPY hydrogel (PHG) beads comprising nanofibrous networks, which were utilized as highly efficient dye absorbents. For instance, PPY hydrogel beads showed a maximum adsorption capacity of 236.9 mg g^−1^ for methyl orange (MO), which was significantly higher than that of PPY powder. The minimal effective concentration of MO for the adsorption was as low as 0.4 ppm. Besides that, the PPY hydrogel beads displayed good regeneration performance for adsorbing organic dyes. Thus, the PPY hydrogel beads with low solid contents and large surface area could be considered as a promising organic dye absorbent for wastewater treatment in various industrial fields.

## Abstract 2

Pure polypyrrole (PPY) hydrogel bead is a type of dye adsorbent with good adsorbing capacity.

## Graphical abstract

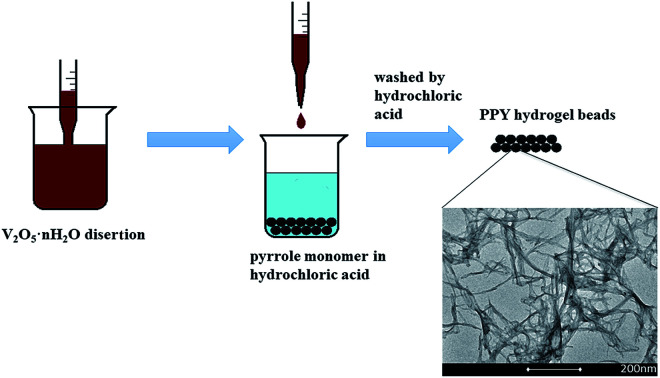

## Introduction

The discharge of organic dyes in various industrial fields such as textiles, plastics, leather, dyestuff and paper-making have been destroying the ecological environment of earth and endangering human health because of their inherent disadvantages of high toxicity, poor biodegradability, carcinogenicity and teratogenicity.^1^ The development of an effective treatment method for removal of organic dyes from wastewater has been attracting a great deal of interest. There are several traditional strategies to remove organic dyes in wastewater, including degradation,^2–5^ coagulation,^6,7^ adsorption,^8–10^ ultrafiltration^11^ and electrophoresis.^12,13^ Among the various strategies, the adsorption-based strategy has drawn intensive attention due to its advantages of a simple operation process, high efficiency, low energy consumption and wide availability. Therefore, adsorption-based strategies for removing organic dyes in wastewater have been widely adopted in various industrial wastewater treatments. As the adsorption-based strategies greatly rely on the dye adsorption capacity and regeneration performance of adsorbents, the development of simple, efficient and environmentally friendly adsorbents has become a research hotspot in chemistry and materials science.

Pristine graphene and its derivatives display very high dye adsorption capacities, but their high cost hinders their practical applications in industrial scale wastewater treatment.^14,15^ Porous carbon materials such activated carbon^16,17^ and multi-walled carbon nanotube^8,18^ are also used as dye adsorbents, but their practical applications are restricted by the low recyclability and complicated preparation processes.

Since Shirakawa *et al.* first reported the synthesis of conductive poly-acetylene,^19^ conductive polymers had formed a new interdisciplinary field, and had shown a wide application prospects in the field of sensor,^20,21^ electromagnetic shielding,^22,23^ supercapacitors^24^ and heavy metal adsorption.^25^ Among various conductive polymers, by virtue of its excellent conductivity, easy synthesis, non-toxic, long-term environmental stability, excellent redox properties, polypyrrole (PPY) has attracted some research interests as a dye adsorbent in recent years.^26–28^ Owing to the existence of positively charged nitrogen atoms on the polymer skeleton, PPY has a certain adsorption capacity through ion exchange and electrostatic interaction.^28^ However, PPY powder does not exhibit high dye adsorption capacity which is mainly attributed to the lack of porous structure and low surface area. In recent years, hydrogels have received tremendous attentions due to their unique geometric and physical–chemical properties in comparison with the corresponding bulk materials. Enlightened by the intrinsic structural merits of hydrogels, it is thus highly desirable to judiciously develop pure PPY hydrogels (PHG) as an efficient organic dye adsorbent. Nevertheless, the shortage of effective methods for preparing pure PHG greatly obstructs their wide applications. Owing to the rigid molecular chains, pristine polymer like PPY are actually insoluble in most of organic solvents and water, which leads to the difficulty of obtaining polymer hydrogels through dissolution–gelation route.^29^ Currently, *in situ* polymerization is considered as a main method to prepare PPY hydrogels. The polymerization is typically performed in another pre-synthesized hydrogel or with the help of other template molecules or nanomaterials, thus the prepared hydrogels normally are composites. Despite the endeavours, rational construction of pure PPY hydrogels with high adsorption capacity and good regeneration performance *via* a facile and green approach still constitutes a longstanding challenge by virtue of the insolubility of PPY in water. In our previous work, an oxidant-templating method had been developed to fabricate pure PANI hydrogels as a supercapacitor electrode which exhibited high specific capacitance and good cycling stability.^30^ However, the fabrication of pure PPY hydrogel and its application as a dye adsorbent in wastewater treatment have not been reported in the opening literature. Besides that, dye adsorbent in powder form easily agglomerate in water and incurs complex post-separation process for recovering adsorbent from water. Herein, a modified one-step method has been developed to fabricate pure PPY hydrogel beads by using vanadium pentoxide (V_2_O_5_) nanowire dispersion as oxidant and sacrificial template. Owing to the large aspect ratio of V_2_O_5_ nanowires, the PPY hydrogel beads can be prepared at very low concentration. Additionally, as sacrificial template, V_2_O_5_ nanowires can be consumed during the *in situ* polymerization of PPY and converted into soluble species, which leads to the formation of pure PPY hydrogel beads after being washed off the soluble species. The obtained pure PPY hydrogel beads exhibited high adsorption capacity of 236.9 mg g^−1^ for methyl orange (MO) and minimal effective concentration of down to 0.4 ppm, which are mainly attributed to the high surface area generated by the unique porous network constructed by interconnected polymer nanofibers. Additionally, the PPY hydrogel beads displayed excellent regeneration performance for adsorbing MO in wastewater.

## Results and discussions

**Fig. 1:**
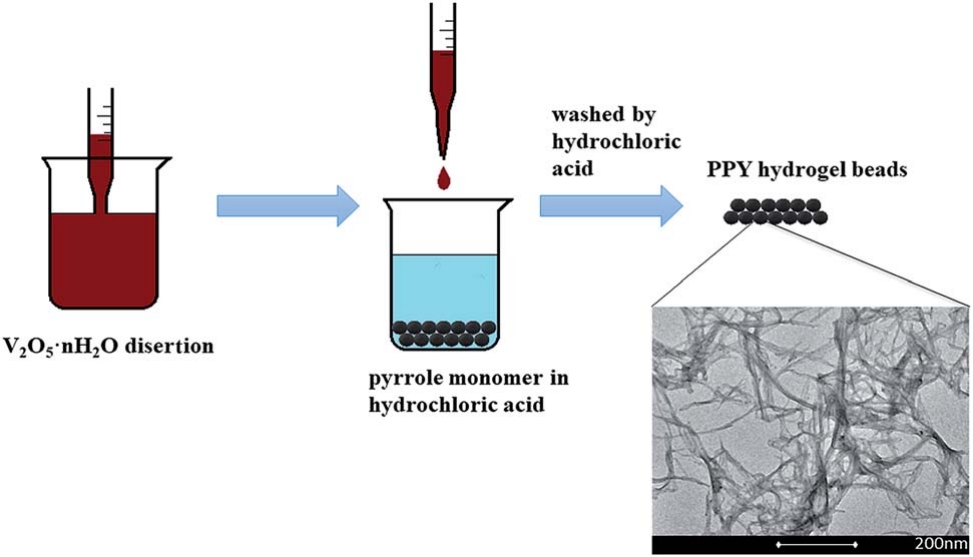
Schematic illustration of the fabrication of the PPY hydrogel beads.

**Fig. 2:**
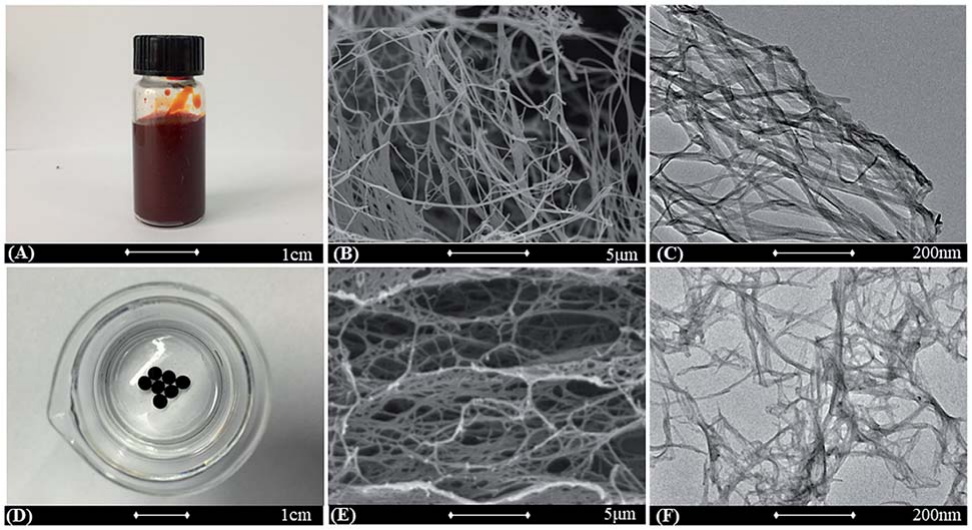
(A) Digital image of the V_2_O_5_ nanowires. (B and C) SEM image (B) and TEM image (C) of V_2_O_5_ nanowires. (D) Digital image of the PHG beads. (E and F) SEM image (E) and TEM image (F) of lyophilized PHG beads.

Fig. 1 shows the fabrication process of the PPY hydrogel beads using V_2_O_5_ nanowires dispersion as oxidant and sacrificial template. Briefly, to promote the adsorption of PPY hydrogel toward organic dye and convenient for recycling, PPY hydrogel beads with sizes of 2.0–4.0 mm were produced by adding the V_2_O_5_ nanowires dispersion into pyrrole monomer in hydrochloric acid dropwisely with a pipette, and the solution gelated within 10 seconds. The V_2_O_5_ dispersion can be obtained by a sol–gel process according to previous work reported by Xiong *et al.*^31^ When contacted with pyrrole solution, the colour of V_2_O_5_ nanowires dispersion was changed from dark red (Fig. 2A) to black (Fig. 2D) in seconds, but more time was needed for the completion of polymerization process.

**Fig. 3:**
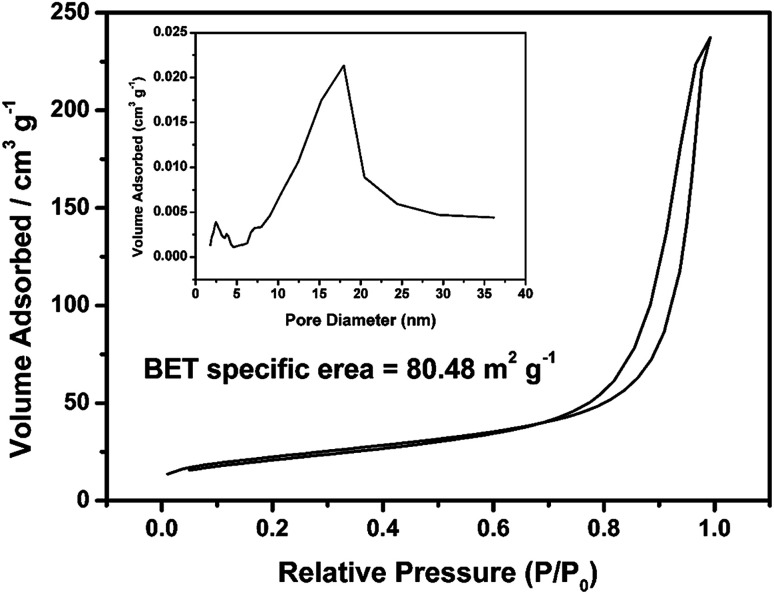
Nitrogen adsorption–desorption isotherm of lyophilized PHG beads; inset shows the plot of pore diameter of PHG beads.

The morphologies of V_2_O_5_ dispersion and lyophilized PHG are investigated using Scanning Electronic Microscopy (SEM) and Transmission Electronic Microscopy (TEM). Both SEM (Fig. 2B) and TEM (Fig. 2C) images show that the morphologies of V_2_O_5_ are ultrathin nanowires with average diameter of 4.0 ± 0.6 nm. The SEM image (Fig. 2E) of lyophilized PHG bead shows that the PPY hydrogel bead has similar interconnected network composed of interconnected nanofibers with extreme aspect ratios. The TEM image of the PPY hydrogel bead is shown in Fig. 2F, it is obviously observed that the average diameter and length of the PPY nanofibers are 3.8 ± 0.8 nm and 0.76 ± 0.2 μm, respectively. The aspect ratio of the obtained PPY nanofibers is around 160–200 which is much larger than that of other previously reported PPY nanofibers.^32,33^ The morphological similarity between the PPY nanofibers (Fig. 2E and F) and V_2_O_5_ nanowires (Fig. 2B and C) demonstrates that the skeleton of the V_2_O_5_ nanowires was well duplicated by the PPY nanofibers. The large aspect ratio and cross-linking network can explain the gelation of the PPY hydrogels at such low solid contents. Moreover, the PPY nanofibers with large aspect ratios and cross-linking network ensure high specific surface area of up to 80.48 m^2^ g^−1^ (Fig. 3) and pore size distribution mainly in the range of 5.0–20.0 nm (Fig. 3 inset). To calculate the solid content of polypyrrole, the hydrogel beads were freezed-dried to remove DI water. Based on calculation, the solid content of polypyrrole in the hydrogel beads is around 1.48%.

**Fig. 4:**
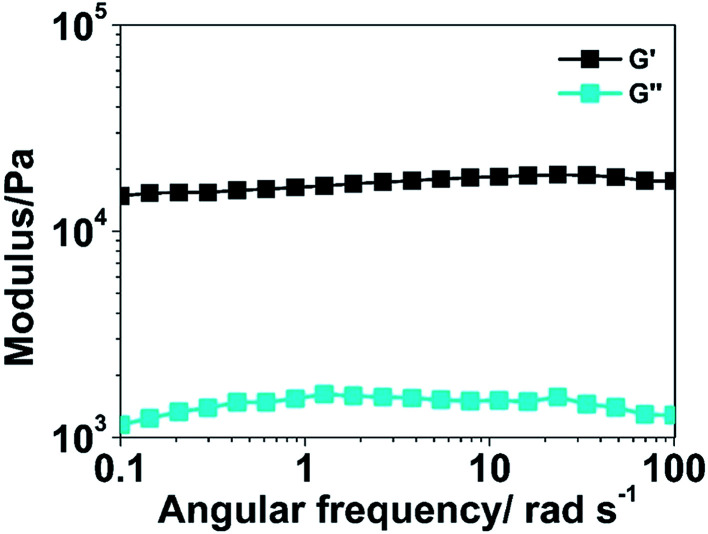
Rheological behaviour of the PHG bead.

The rheological profiles of PHG beads are shown in Fig. 4. The storage modulus is approximately an order of magnitude higher than the loss one at the angular frequencies of 1–100 rad s^−1^, which indicates that the elastic response is predominant in the permanent network. The high storage modulus (∼10^4^) proves the strong mechanical properties of the PPY hydrogel beads as well.

**Fig. 5:**
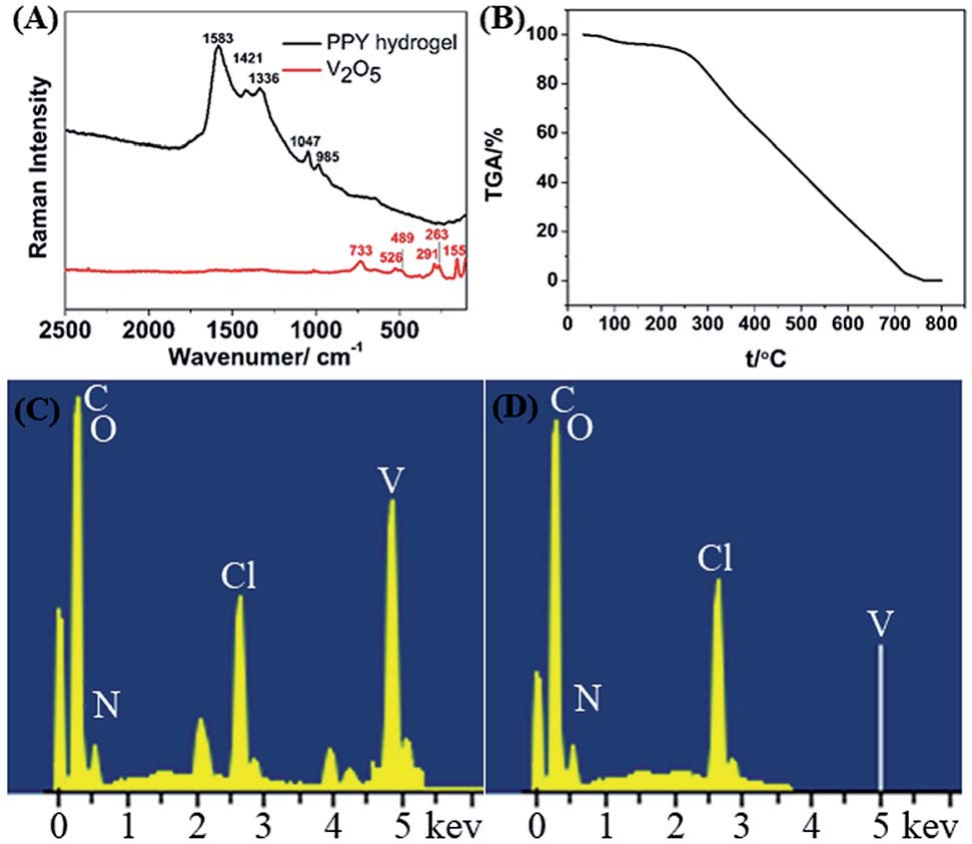
(A) Raman spectra of the lyophilized PHG beads and V_2_O_5_ nanowires. (B) TGA curves of the lyophilized PHG beads. (C and D) EDS spectrum of lyophilized PHG beads before and after being washed by hydrochloric acid and DI water.

The PPY hydrogel bead was analysed by using Raman spectrum with excitation wavelength of 532 nm. As shown in Fig. 5A, the Raman spectrum exhibits five characteristic peaks at 985, 1047, 1336, 1421 and 1583 cm^−1^. The peaks at 1583 cm^−1^ and 1421 cm^−1^ are assigned to the C=C and C–N stretching vibration, respectively. The broad peak observed at 1047 cm^−1^ corresponds to the C–H in-plane deformation and the small peak at 985 cm^−1^ is attributed to the bipolaronic structure.^34^ These characteristic peaks confirm that the *in situ* oxidation polymerization of pyrrole leads to the formation of PPY. On the other hand, characteristic peaks belonging to the V_2_O_5_ are not observed, which implies that V_2_O_5_ is completely removed during the oxidation and washing process.^35^ To further prove the V element does not exist in the PHG beads, both thermos gravimetric analysis (TGA) and energy dispersive X-ray spectrum (EDS) are conducted. The TGA curve in Fig. 5B shows that the residual weight of PHG beads at 730 °C is approximately 0.082%, suggesting that there is no non-volatile inorganic component in the PHG beads. Additionally, the energy dispersive spectrum (EDS) in Fig. 5C and D reveal that characteristic peak attributing to V element is disappeared after the PHG beads being washed by hydrochloric acid and DI water. Based on the above Raman, TGA and EDS results, it is obviously that the PHG beads are composed of pure PPY.

**Fig. 6:**
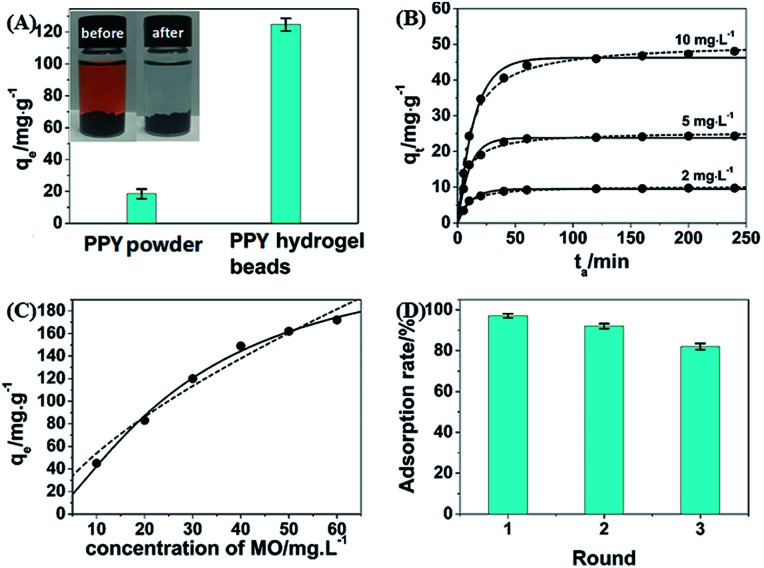
(A) The adsorption capabilities of PPY powder and the PHG beads toward MO solution (30 mg L^−1^). Inset shows the image of 20 mL MO solution (30 mg L^−1^) before (left) and after (right) being absorbed by the PHG beads. (B) Plots of the adsorption uptake (*q*_*t*_) of MO by the PHG beads *versus* adsorption time (*t*_a_). The PHG beads are incubated in 20 mL of the MO solution for adsorption and the initial MO concentration of the organic dyes is varied from 2 to 5 and 10 mg L^−1^. The adsorption kinetics profiles (open squares) are fitted by pseudo-first-order (solid curves) and pseudo-second-order models (dashed curves), respectively. (C) Adsorption isotherms of MO (open squares) by the PHG beads, which are fitted by Langmuir (solid curves) and Freundlich models (dashed curves), respectively. (D) The adsorption capacity of the PHG beads after 3 times of cycle experiments.

To evaluate the dye adsorbent capacity of the obtained PHG beads, MO was used as dye model and the PHG beads were incubated in MO solution at a concentration of 30 mg L^−1^. After being incubated for 4 h, the MO solution becomes colourless and transparent (Fig. 6A inset) indicating efficient removal of MO molecules from water. Fig. 6A shows that the MO adsorption capacity of the PHG beads is 122.4 mg g^−1^, which is near 7 times higher than that of PPY powder (18.4 mg g^−1^) under the same conditions. The significant enhanced adsorption capacity of the PHG beads can be mainly attributed to the high surface area, unique porous and interconnected nanostructure.

Fig. 6B shows the adsorption kinetics of the PHG beads at different incubation time using different concentrations (2, 5 and 10 mg L^−1^) of MO solution as dye model. The adsorption kinetics are fitted better by the pseudo-second-order model than the pseudo-first-order model, as substantiated by the coefficient of determination (*R*^2^) listed in Table S1,† which suggests that chemisorption serves as the main-determining step for the adsorption of MO.^36^ The hydrogen bonding between the MO dye and PPY, as drawn in Schemes S1 and S2,† should be the driving force for adsorption of MO dye by the PHG beads. Fig. 6C shows the adsorption isotherm of the PHG beads towards MO solution and the analysed results are listed in Table S2.† Table S2† suggests that Langmuir model fits the adsorption isotherms better than Freundlich model and the value of 1/*n* < 1 leads to poor fitting results,^37^ which confirm homogeneous monolayer adsorption of the MO molecules by the PHG beads. Table S2† also reveals that the maximum adsorption capacity (*q*_m_) of the PHG beads towards MO is estimated to be 236.9 mg g^−1^. Adsorption capacities of some adsorbents reported in previous works are listed in Table S3† and compared with our current work. As shown in Table S3,† the adsorption capacity of the PHG beads is higher than most of the other previously reported adsorbents. This suggests that the PHG bead is an effective adsorbent towards MO. Moreover, Fig. S1A† reveals that the PHG beads are able to adsorb MO at very low concentration of down to 0.4 ppm. As shown in Fig. S1B,† further decreases the concentration of MO to 0.3 ppm, no obvious MO dye adsorption can be observed. In addition, the regeneration ability of the PHG beads as a dye adsorbent is also investigated and the results are shown in Fig. 6D. Obviously, the adsorption capacity of the PHG beads can maintain more than 80% of the initial adsorption capacity after 3 times of cycle experiments, which indicate that the PHG beads probably can be considered as a re-generable dye adsorbent.

**Fig. 7:**
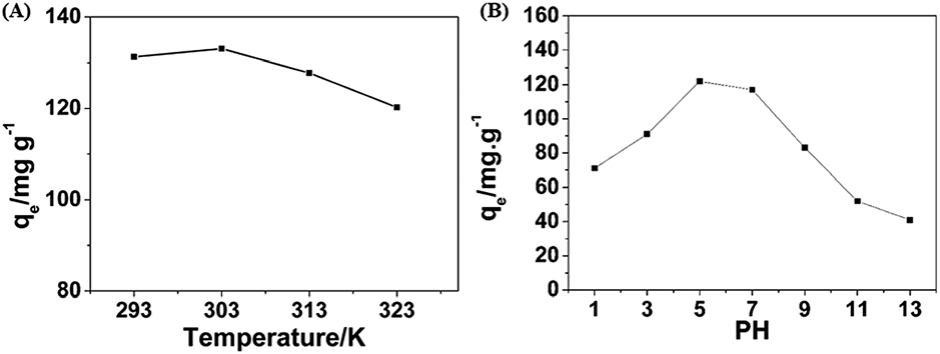
(A) Plots of the adsorption uptake (*q*_e_) of MO by the PHG beads *versus* different temperature. (B) Plots of the adsorption uptake (*q*_e_) of MO by the PHG beads *versus* different pH value.

In order to discuss the effect of temperature on the adsorption performance, a series of experiments in the range of 293–323 K were conduced and results are shown in Fig. 7A. With the increase of temperature, the adsorption capacity of the PHG beads showed a slight decreasing trend, which is due to the fact that this adsorption is an exothermic phenomenon and the increase of temperature is not conductive to adsorption.^38^

The adsorption capacities of the PHG beads under different pH values are shown in Fig. 7B. The adsorption capacities are maintained effective in weakly acidic range between pH = 5–7, while the adsorption capacity decreases when pH < 5 or pH > 7. This adsorption behaviour can be explained as follow. MO presents in cationic form in acidic medium (pH < 7) due to banding of H^+^ with –SO_3_^−^ to form –SO_3_H, while presents in anionic form in basic medium.^39^ With the further decrease of pH value (pH < 5), the electrostatic repulsion force between the cationic form of MO and the positive charge of the adsorption site is enhanced which leads to the decrease of adsorption. As shown in Fig. S2,† zeta potential of the PHG beads decreases from 23.96 mV to −3.67 mV with the increase of pH value in range of 3–11, and the neutral point is between the pH value of 9 and 10. This result demonstrates that with the increase of pH value (pH > 7), the competitive interaction between excess OH^−^ and anions of the MO molecule on the adsorption site of the PHG beads and the deprotonation of the nitrogen atoms in the PHG matrix leading to the decrease of adsorption capacity at higher pH values.^40^

**Fig. 8:**
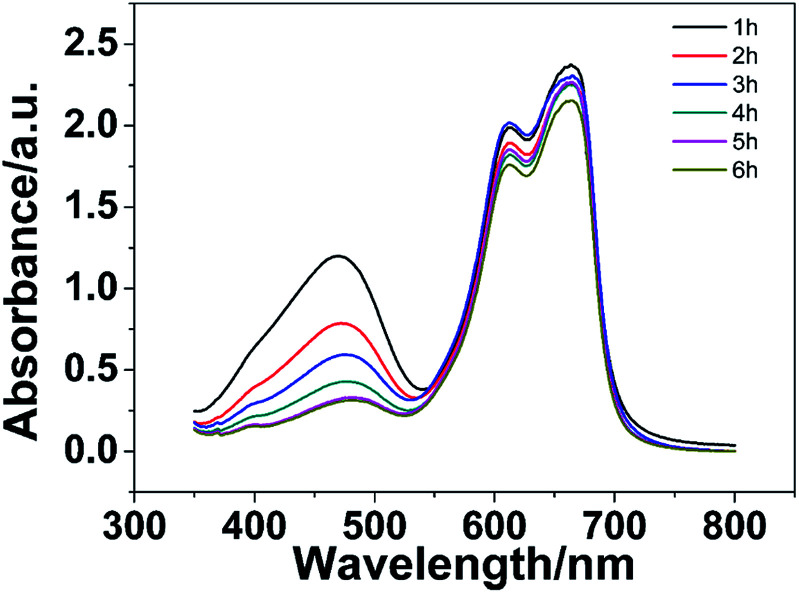
The UV-vis spectra of MO/MB solution (30 mg L^−1^) adsorbed by the PHG beads at different time during the adsorption experiment.

Moreover, the selective adsorption performances of the PHG beads were also investigated by using methylene blue (MB), MO and mixture of MO/MB as dye models. The digital images of MB, MO and MO/MB solution before and after being absorbed by the PHG beads are shown in Fig. S3.† No obvious difference between the colour of MB solution before and after adsorption is observed, indicating that the PHG beads cannot adsorb MB effectively. However, the colour MO solution is transparent and colourless after being adsorbed by the PHG beads, which proves that the PHG beads can adsorb MO dye effectively. Regarding the MO/MB mixture solution, the colour of mixture solution is changed from black to blue after being adsorbed by the PHG beads, which reveals that the MO molecules in the mixture solution is efficiently adsorbed whereas no obvious MB molecules is adsorbed by the PHG beads. UV-vis spectrum of the MO/MB mixture solution was also measured to investigate selective adsorption performance of the PHG beads and the results are shown in Fig. 8. The peak intensity of MO at 463 nm gradually decreases with the increase of incubation time, which indicates that MO molecules are efficiently adsorbed by the PHG beads. However, the peak intensities of MB at 615 and 653 nm are only slightly decreased even after 6 h. Both of the digital images and UV-vis spectrum prove that the PHG beads prefer to adsorb MO molecules instead of MB molecules. As shown in Schemes S1 and S2,† the existence of similar positively charged nitrogen atoms causes a large electrostatic repulsion between MB molecules and the PHG beads, which leads to the poor adsorption performance.

## Conclusions

In summary, an oxidant-templating method using V_2_O_5_ nanowires as oxidant and sacrificial template has been developed to fabricate pure PPY hydrogel beads as a highly efficient dye adsorbent, especially for MO dye. The PHG beads show a maximum adsorption capacity of 236.9 mg g^−1^ for MO, which is much higher than that of PPY powder and most of the previously reported adsorbents. The minimal effective concentration of MO dye is as low as 0.4 ppm. Additionally, the PHG beads maintain more than 80% adsorption capacity after 3 times cycle experiments. The optimum temperature and pH value for adsorbing MO are 303 K and pH = 5, respectively. Meanwhile, the PHG beads can be considered as selective adsorbent for negative charged dyes, such as MO.

## Experimental section

### Preparation of V_2_O_5_ dispersion

The synthesis method was based on previous work reported by Xiong *et al.*^31^ 1.0 g of NH_4_VO_3_ was grinded with few drops of DI water in a bowl of agate stone. The grinding fluid was then mixed with 10 mL of 1 M HCl solution under continuous stirring. Afterward, as the mixture became red, DI water was added to reach a total volume of 20 mL. After sedimentation of the red precipitate, the supernatant was removed. The resulting red precipitate was dispersed in hot water (90 °C) with a total volume of 20 mL. After vigorous stirring, the supernatant was removed. This process was repeated three times and then the red dispersion was refilled with hot water to a total volume of 40 mL.

### Preparation of PPY hydrogel beads

The obtained V_2_O_5_ dispersion was dropwisely added into the mixture of 0.3 mL pyrrole and 39.7 mL DI water to form PPY hydrogel beads. To remove excess acid and by-products from polymerization, the PPY hydrogel was purified by 1 M HCl (100 mL) and large amounts of DI water.

### Characterizations

The morphologies of the PPY hydrogels were observed by a SU-70 scanning electron microscopy (Hitachi, Japan) at an accelerating voltage of 20 kV equipped with an energy dispersive X-ray analyzer. The morphology of V_2_O_5_ nanowires was observed on JEM-2100 transmission electron microscopy (JEOL, Japan) at 200 kV. Rheology measurements were carried out by using a MCR 302 Rhometer (Anton Paar, Austria). Raman spectra were recorded at room temperature with a MicroRaman System RM3000 spectrometer and an argon ion laser operating at a wavelength of 532 nm as the excitation laser. Thermogravimetric analysis (TGA) was carried out on a STA 449 F3 Jupiter simultaneous thermal analyzer with a heating rate 20 °C min^−1^ in the air condition. Zeta potential was determined by electrophoretic light scattering (ELS) with Zetaplus (Brookhaven Instruments Corporation, Holtsville, NY, USA). BET surface area was measured by TriStar 3000 specific surface area analyzer.

### Adsorption kinetics of the PPY hydrogel beads^41^

Organic dye (MO) was used to evaluate the adsorption and cycling performances of the as-prepared PPY hydrogel beads. A series of batch equilibrium adsorption experiments were carried out at room temperature. The temporal evolution of the dye concentration was analyzed with the help of UV-vis spectroscopy. The adsorption capacity (*q*_*t*_, mg g^−1^) of the PPY hydrogel beads can be correlated with the adsorption time (*t*_a_, min) as below:1
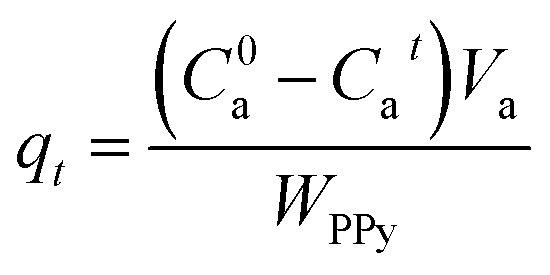
where *C*^0^_a_ (mg L^−1^) is the initial concentration of the organic dye, *C*_a_^*t*^ (mg L^−1^) is the concentration of organic dye at a given *t*_a_, *V*_a_ (mL) is the total volume of the solution of the organic dye (here *V*_a_ = 20 mL), and *W*_PPY_ (g) is the mass of the PPY hydrogel beads. For all the adsorption and cycling experiments, the initial concentration of organic dyes (*C*^0^_a_) was varied from 60 to 30, 20, 10, 5, and 2 ppm. After the adsorption for a given time (*t*_a_), the concentration of the organic dyes remained in solution (*C*_a_^*t*^) was assessed by UV-vis spectroscopy. The values of *q*_*t*_, calculated according to eqn (1), were plotted as a function of *t*_a_ to determine the adsorption kinetics model.

When the equilibrium adsorption was reached, the equilibrium adsorption capacity (*q*_e_, mg g^−1^) of the PPY hydrogel beads can be correlated with the equilibrium concentration (*C*^e^_a_) of the organic dyes remained in solution as below:2
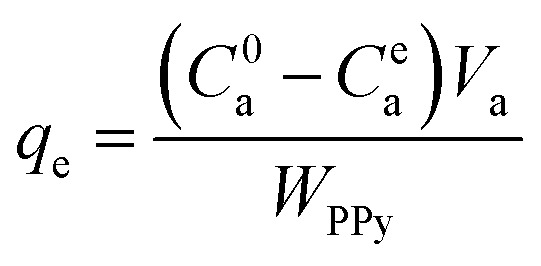


The adsorption isotherms were obtained by plotting the calculated values of *q*_e_ as a function of the experimentally measured values of *C*^e^_a_, from which the adsorption thermodynamic model and the maximum adsorption capacity were determined.

### Evaluation of the regeneration ability of the PPY hydrogel beads

After the adsorption process, the PPY hydrogel beads were collected and immersed in ethanol for 1 h to desorb organic dye. Then the adsorption–desorption process was repeated two more times. The adsorption capacities of each cycle were measured by UV-vis spectroscopy.

## Conflicts of interest

There are no conflicts to declare.

## Supplementary Material

RA-009-C9RA00209J-s001

## References

[cit1] Brillas E., Martínez-Huitle C. A. (2015). Appl. Catal., B.

[cit2] Nath K., Chandra M., Pradhan D., Biradha K. (2018). ACS Appl. Mater. Interfaces.

[cit3] Wang J., Bai R. (2016). Water Res..

[cit4] Ge L., Peng Z., Wang W., Tan F., Wang X., Su B., Qiao X., Wong P. K. (2018). J. Mater. Chem..

[cit5] Zhou C., Luo J., Chen Q., Jiang Y., Dong X., Cui F. (2015). Chem. Commun..

[cit6] Han G., Liang C.-Z., Chung T.-S., Weber M., Staudt C., Maletzko C. (2016). Water Res..

[cit7] Jeon S.-B., Kim S., Park S.-J., Seol M.-L., Kim D., Chang Y. K., Choi Y.-K. (2016). Nano Energy.

[cit8] Luan J., Hou P.-X., Liu C., Shi C., Li G.-X., Cheng H.-M. (2016). J. Mater. Chem..

[cit9] Basak S., Nandi N., Paul S., Hamley I. W., Banerjee A. (2017). Chem. Commun..

[cit10] Yu D., Wang H., Yang J., Niu Z., Lu H., Yang Y., Cheng L., Guo L. (2017). ACS Appl. Mater. Interfaces.

[cit11] Wolf T., Niazov-Elkan A., Sui X., Weissman H., Bronshtein I., Raphael M., Wagner H. D., Rybtchinski B. (2018). J. Am. Chem. Soc..

[cit12] Zhang Z., Zhu L., Lu W., Li X., Sun X., Lü R., Ding H. (2016). Chem. Eng. Res. Des..

[cit13] Fan L., Ni J., Wu Y., Zhang Y. (2009). J. Hazard. Mater..

[cit14] Zhang X., Liu D., Yang L., Zhou L., You T. (2015). J. Mater. Chem..

[cit15] Qi Y., Yang M., Xu W., He S., Men Y. (2017). J. Colloid Interface Sci..

[cit16] Asfaram A., Ghaedi M., Hajati S., Goudarzi A. (2015). RSC Adv..

[cit17] Saucier C., Adebayo M. A., Lima E. C., Cataluña R., Thue P. S., Prola L. D., Puchana-Rosero M., Machado F. M., Pavan F. A., Dotto G. (2015). J. Hazard. Mater..

[cit18] Apul O. G., Karanfil T. (2015). Water Res..

[cit19] Shirakawa H., Louis E. J., MacDiarmid A. G., Chiang C. K., Heeger A. J. (1977). J. Chem. Soc., Chem. Commun..

[cit20] Maeda K., Hirose D., Okoshi N., Shimomura K., Wada Y., Ikai T., Kanoh S., Yashima E. (2018). J. Am. Chem. Soc..

[cit21] Zeng Q., Cai P., Li Z., Qin J., Tang B. Z. (2008). Chem. Commun..

[cit22] Zhao H., Hou L., Lu Y. (2016). Chem. Eng. J..

[cit23] Yan D. X., Pang H., Li B., Vajtai R., Xu L., Ren P. G., Wang J. H., Li Z. M. (2015). Adv. Funct. Mater..

[cit24] Wang K., Wu H., Meng Y., Wei Z. (2014). Small.

[cit25] Karthik R., Meenakshi S. (2015). Chem. Eng. J..

[cit26] Xin S., Yang N., Gao F., Zhao J., Li L., Teng C. (2017). Appl. Surf. Sci..

[cit27] Li X., Lu H., Zhang Y., He F. (2017). Chem. Eng. J..

[cit28] Haque M. M., Smith W. T., Wong D. K. (2015). J. Hazard. Mater..

[cit29] Liu S., Wang F., Dong R., Zhang T., Zhang J., Zhuang X., Mai Y., Feng X. (2016). Adv. Mater..

[cit30] Zhou K., He Y., Xu Q., Zhang Q. e., Zhou A. a., Lu Z., Yang L.-K., Jiang Y., Ge D., Liu X. Y. (2018). ACS Nano.

[cit31] Xiong C., Aliev A. E., Gnade B., Balkus Jr K. J. (2008). ACS Nano.

[cit32] Zhang X., Manohar S. K. (2004). J. Am. Chem. Soc..

[cit33] Wang Y., Shi Y., Pan L., Ding Y., Zhao Y., Li Y., Shi Y., Yu G. (2015). Nano Lett..

[cit34] Biswas S., Drzal L. T. (2010). Chem. Mater..

[cit35] Peng X., Zhang X., Wang L., Hu L., Cheng S. H. S., Huang C., Gao B., Ma F., Huo K., Chu P. K. (2016). Adv. Funct. Mater..

[cit36] Ho Y. S., Ng J. C. Y., McKay G. (2000). Sep. Purif. Methods.

[cit37] Iqbal J., Wattoo F. H., Wattoo M. H. S., Malik R., Tirmizi S. A., Imran M., Ghangro A. B. (2011). Arabian J. Chem..

[cit38] Saffarionpour S., Tam S.-Y. S., Van der Wielen L. A. M., Brouwer E., Ottens M. (2019). Sep. Purif. Technol..

[cit39] Darwish A. A. A., Rashad M., Al-Aoh H. A. (2019). Dyes Pigm..

[cit40] Xin Q., Fu J., Chen Z., Liu S., Yan Y., Zhang J., Xu Q. (2015). J. Environ. Chem. Eng..

[cit41] Li Y., Xiaokong L., Weichang Y., Lauren Joan B., Dayang W. (2015). Langmuir.

