# Supplementary material for: Oxidant-templating fabrication of pure polypyrrole hydrogel beads as a highly efficient dye adsorbent
Source: RSC Adv. 2019 Feb 18;9(11):5895–900. doi: 10.1039/c9ra00209j (PMC9060859; doi:10.1039/c9ra00209j)
Supplement: RA-009-C9RA00209J-s001 [file RA-009-C9RA00209J-s001.pdf]

Electronic Supplementary Material (ESI) for RSC Advances.
This journal is © The Royal Society of Chemistry 2019

Supporting Information

Oxidant-Templating Fabrication of Pure Polypyrrole Hydrogel Beads as Highly Efficient Dye Adsorbent

Xi Ye[a], Qingchi Xu[ab*], Jun Xu[ab*]

[a] Department of Physics, Research Institute for Biomimetics and Soft Matter, Fujian Provincial Key Laboratory for Soft Functional Materials, Xiamen University, Xiamen, 361005, P. R. China.

[b]Jiujiang Research Institute of Xiamen University, Jiujiang 332000, P. R. China.

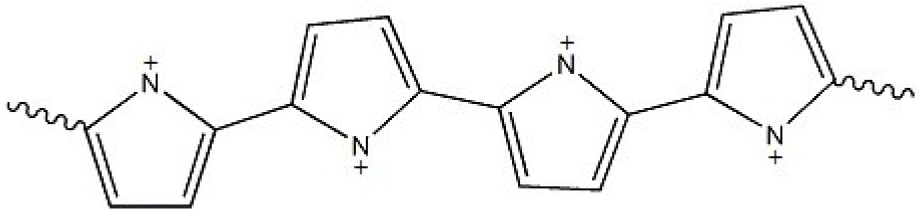

Scheme. S1 Chemical structure of polypyrrole.

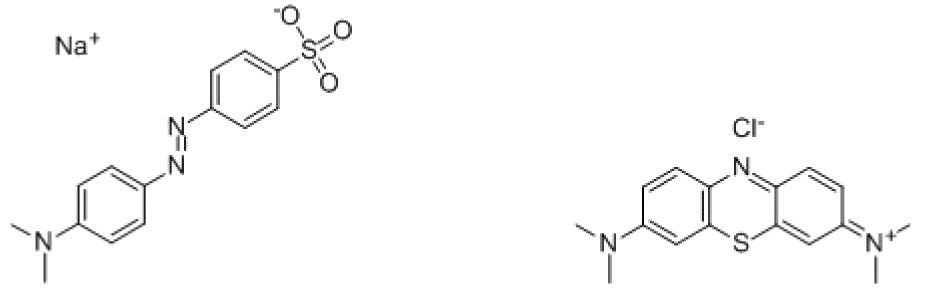

Scheme.S2 Chemical structure of methyl orange (MO) and methylene blue (MB).

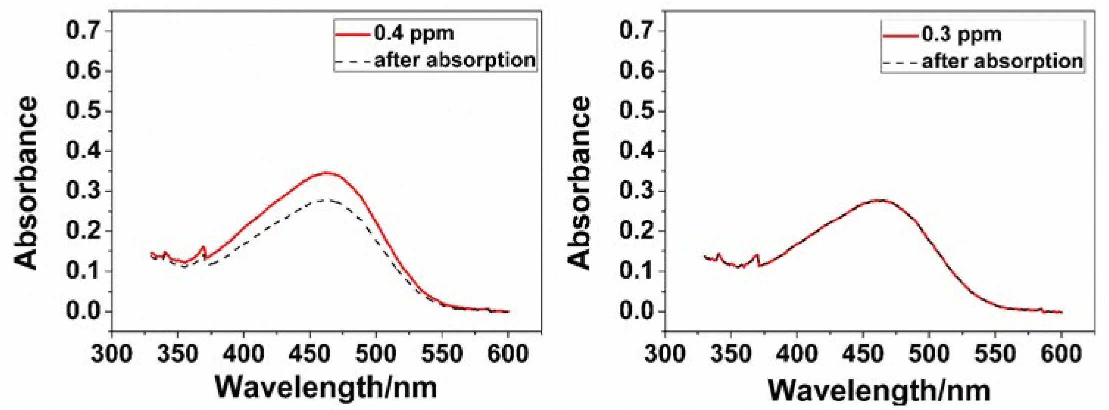

Fig. S1 UV-vis spectra of the MO solution before (dashed curves) and after (solid curves) absorption using PHG beads at the MO concentration of 0.4 ppm (left) and 0.3 ppm (right).

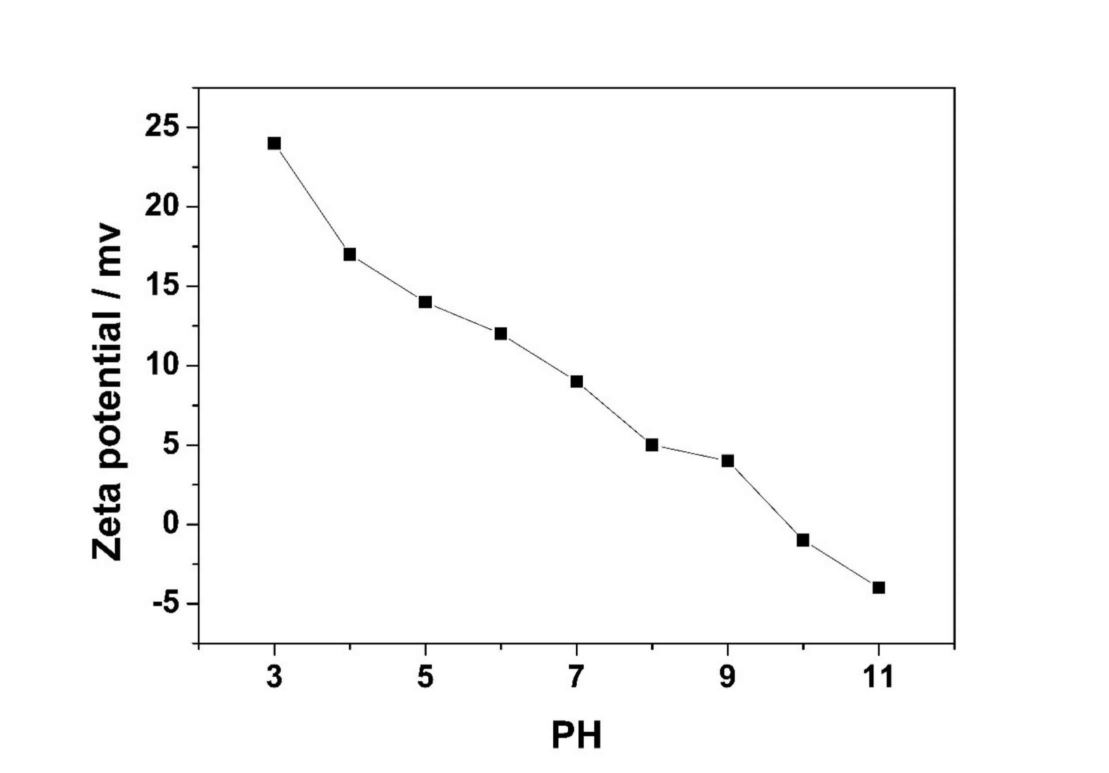

Fig. S2 Zeta potential of the PHG beads

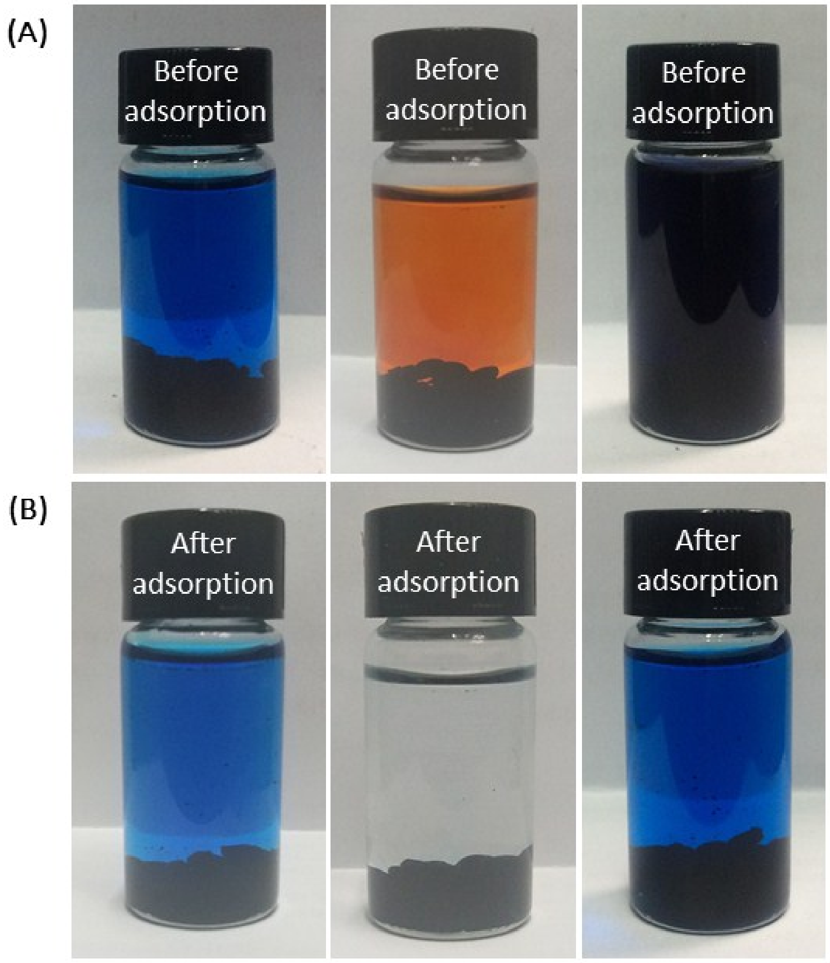

Fig. S3 The photographs of MB, MO, and MB/MO mixture solution before (A) and after (B) adsorption by the PHG beads.

Table S1. Summary of the adsorption kinetics the PHG beads using MO as dye model and the fitting results obtained based on Pseudo-first-order and Pseudo-second-order kinetics models.

		Pseudo first order			Pseudo second order		
C_a^0 (mg g$^{-1)}$	$q_{e\ exp}$ (mg g^{-1})	$q_{e\ cal}$ (mg g^{-1})	K_1 (min^{-1})	R^2	$q_{e\ cal}$ (mg g^{-1})	K_2(g mg^{-1} min^{-1})	R^2
2	9.74	9.49	0.0916	0.977	10.21	0.01299	0.979
5	24.40	23.81	0.1002	0.972	25.46	0.00589	0.975
10	48.84	46.23	0.0689	0.984	50.48	0.00187	0.988

Table S2. Summary of the adsorption isotherms of the PHG beads using MO as dye model based on Langmuir and Freundlich models.

Langmuir model			Freundlich model		
q_m (mg g^{-1})	k_L (L mg^{-1})	R^2	K_F (mg $g^{1-1/n}$ g^{-1} $L^{1/n}$)	1/n (g L^{-1})	R^2
236.9	0.081	0.998	11.3	0.67	0.963

Table S3. Comparison of the adsorption capacities of several adsorbents using MO as dye model

Adsorbents	Adsorption capacity (mg g^{-1})	Ref.
Muli-walled CNT	35.4	1
Activated carbon	11.2	2
MIL-53	57.9	3
PED-MIL-101	194	3
NH_2-MIL-101 (Al)	188	4
UiO-66	73.5	5
PPy nanofibers	169.55	6
PPY hydrogel heads	**236.9**	This work

Reference

1. B. Tanhaei, A. Ayati, M. Lahtinen and M. Sillanpää, *Chem. Eng. J.*, 2015, **259**, 1-10.
2. Y. Yao, B. He, F. Xu and X. Chen, *Chem. Eng. J.*, 2011, **170**, 82-89.
3. E. Haque, J. W. Jun and S. H. Jhung, *J. Hazard. Mater.*, 2011, **185**, 507-511.
4. E. Haque, J. E. Lee, I. T. Jang, Y. K. Hwang, J. S. Chang, J. Jegal and S. H. Jhung, *J. Hazard. Mater.*, 2010, **181**, 535-542.
5. E. Haque, V. Lo, A. I. Minett, A. T. Harris and T. L. Church, *J. Mater. Chem*, 2013, **2**, 193-203.
6. Q. Xin, J. Fu, Z. Chen, S. Liu, Y. Yan, J. Zhang and Q. Xu, *Journal of Environmental Chemical Engineering*, 2015, **3**, 1637-1647.
